# Multiple host colonization and differential expansion of multidrug-resistant ST25-*Acinetobacter baumannii* clades

**DOI:** 10.1038/s41598-023-49268-x

**Published:** 2023-12-09

**Authors:** Agnese Lupo, Benoît Valot, Estelle Saras, Antoine Drapeau, Marine Robert, Maxime Bour, Marisa Haenni, Patrick Plésiat, Jean-Yves Madec, Anaïs Potron

**Affiliations:** 1 Unité Antibiorésistance et Virulence Bactériennes, ANSES - Université de Lyon 1, 31 Avenue Tony Garnier, 69007 Lyon, France; 2 UMR 6249 Chrono-Environnement, CNRS-Université de Bourgogne/Franche-Comté, Besançon, France; 3 CNR de la Résistance aux Antibiotiques, Centre Hospitalier Universitaire de Besançon, Besançon, France

**Keywords:** Evolution, Microbiology

## Abstract

The *Acinetobacter baumannii* clonal lineage ST25 has been identified in humans and animals and found associated with outbreaks globally. To highlight possible similarities among ST25 *A. baumannii* of animal and human origins and to gather clues on the dissemination and evolution of the ST25 lineage, we conducted a phylogenetic analysis on *n* = 106 human and *n* = 35 animal *A. baumannii* ST25 genomes, including 44 sequenced for this study. Resistance genes and their genetic background were analyzed, as well. ST25 genomes are clustered into four clades: two are widespread in South America, while the other two are largely distributed in Europe, Asia and America. One particular clade was found to include the most recent strains and the highest number of acquired antibiotic resistance genes. OXA-23-type carbapenemase was the most common. Other resistance genes such as *bla*_NDM-1_, *bla*_PER-7_, and *arm*A were found embedded in complex chromosomal regions present in human isolates. Genomic similarity among multidrug resistant ST25 isolates of either animal or human origin was revealed, suggesting cross-contaminations between the two sectors. Tracking the clonal complex ST25 between humans and animals should provide new insights into the mode of dissemination of these bacteria, and should help defining strategies for preserving global health.

## Introduction

Multidrug resistant strains of *Acinetobacter baumannii* are a major cause of hard-to-treat healthcare-associated infections worldwide^1^. In particular, the development of carbapenem-resistance, which is mostly associated with the acquisition of β-lactamase-encoding (carbapenemase) genes has gradually become a dramatic medical concern in many hospitals, leaving clinicians with very limited options to treat severely ill patients^2^. The majority of multidrug resistant strains of *A. baumannii* belong to a few clonal lineages, such as international clones IC1 and IC2^3^. However, some reports dating back almost 20 years have described nosocomial outbreaks due to ST25 (belonging to IC7)^4^ *A. baumannii* producing the carbapenemase OXA-23, in Italy, Greece, and Turkey^5^. Since then, multiple resistant ST25 isolates have been identified in various countries, including those where the prevalence of antimicrobial resistance (AMR) is generally low, such as Sweden^6^. The ST25 lineage has also been traced in places where multidrug resistant *A*. *baumannii* are endemic, like South America^7–9^, and East Africa^10–12^. More recently, investigations from Ukraine^13^, Thailand^14^, Australia^15,16^, and France^3^ provided additional data about the expansion of this clonal complex, which is associated with high mortality rates among infected patients^17^.

Over the years, non-human reservoirs of *A*. *baumannii* have been increasingly recognized^18,19^, including companion animals infected with carbapenem-resistant strains^20–27^. Most often, these strains are related to widespread or emerging clones found in human patients, such as ST2. Considering the global distribution of ST2 isolates in hospitals, the possibility of human to animal transmission has been raised^28,29^. In France, continuous surveillance of AMR in diseased animals has highlighted the presence of ST25 *A*. *baumannii* carrying the *bla*_OXA-23_ gene in dogs and cats with urinary tract infections^30^. More surprisingly, this clone was also identified in healthy pets, with no history of antibiotic therapy or admission to veterinary clinic^31^.

Because of the recurrent isolation of ST25 *A*. *baumannii* isolates in animals in France, a more in-depth analysis of these bacteria by whole-genome sequencing was undertaken. Then, in order to elucidate possible links between the animal and human sectors, we included in the analysis human strains collected in France during the same period of time as well as all ST25 *A*. *baumannii* genomes available in open databases.

## Materials and methods

### Description of the ST25 collection

In the period 2010–2019, 341 isolates of *Acinetobacter* spp. that were collected by the veterinary diagnostic laboratories participating in French network Resapath (https://resapath.anses.fr/), were referred to ANSES-Lyon laboratory. There, the strains were identified at the species level by gene *rpoB* sequencing, and their sequence type (ST) was determined according to the Pasteur Institute scheme^32^. One hundred and eighty-five of these were found to belong to the species *A*. *baumannii*, of which 42 could be assigned to ST25. Only non-redundant ST25 isolates originating from single animals (*n* = 33) were retained for the present study. The collection was subsequently enriched with 11 ST25 strains of human origin collected by the French National Reference Center for Antibiotic Resistance (NRC-AR, Besançon hospital, France) between 2013 and 2018 (Supplementary Table S1).

### Antibiotic susceptibility testing

Susceptibility of the ST25 *A*. *baumannii* strains to 12 antibiotics (amikacin 30 µg, gentamicin 10 µg, tobramycin 10 µg, ciprofloxacin 5 µg, piperacillin 30 µg, piperacillin 30 µg + tazobactam 6 µg, ticarcillin 75 µg, ticarcillin 75 µg + clavulanic acid 10 µg, imipenem 10 µg, meropenem 10 µg, ceftazidime 10 µg, cefepime 30 µg) was assessed by the disk diffusion method (Mast Diagnostic, France) on Mueller Hinton agar (Biorad, France) according to the guidelines and clinical breakpoints (human and veterinary) of the Antibiogram Committee of the French Society for Microbiology^33,34^. *Pseudomonas aeruginosa* reference strain ATCC 27853 was used as a quality control.

### Whole-genome sequencing experiments

The whole DNA of the 44 selected strains was extracted (NucleoSpin Microbial DNA, Macherey–Nagel, Germany), and then quantified spectrophotometrically (NanoDrop, Thermo Fisher Scientific, France) and by fluorometry (Qubit, dsDNA BR Assay Kit, Life Technologies, USA). Preparation of DNA libraries and whole-genome sequencing experiments were out-sourced to services using a NovaSeq6000 Illumina platform generating paired-end reads (Institut du Cerveau et de la Moelle Epinière, Paris). Long-reads sequencing experiments were also performed by using the Oxford Nanopore technology, on a MinION sequencer equipped with a R9.4.1 flow cell, for 10 isolates (38208, 39518, 43344, 46732, 48031, 48427, 51877, 13A462, 14A543, 15A1044) differing in their antibiotic resistance gene (ARG) content and their host origin. Sequences are available from the Bioprojects PRJNA838428 and PRJNA766794 (https://www.ncbi.nlm.nih.gov/bioproject).

### Bioinformatic analyses

Base calling for long-reads sequencing was performed using Guppy 3.4.5. Short reads were assembled using Shovill v.1.0.4 with the default option. Long-short reads hybrid assembly was obtained using Unicycler v.0.4.8^35^. The quality and assembly of the reads were verified by FastQC v.0.11.9 and Quast v.5.0.2^36^ (Supplementary Table S1). Gene annotation was done using RASTtk^37^ on Patric 3.6.9 platform. Genes conferring antibiotic resistance were searched using ResFinder 4.1^38^. The in silico resistance search was performed with a minimum coverage of 80% and a minimum identity of 90%. For each resistance gene detected in a strain, only the best alignment was reported, when different variants were matching.

Our phylogenetic analysis of ST25 strains was carried out on the 44 genomes mentioned above, as well as ST25 genomes and single locus variants (ST25 SLVs) available from the repository of National Center of Biology Information (NCBI). The database, last accessed in January 2022, contained 5837 *A. baumannii* genome sequences, of which 162 (2.8%) were from ST25 strains and 24 (0.4%) from ST25 SLVs. Their core-genome analysis was undertaken (https://github.com/bvalot/pyMLST) based on 2,386 genes preselected from *A*. *baumannii* (cgmlst.org). The analysis was conducted using MrBayes-3.2.7 with GTR + G + I models^39^. Analysis was performed with 10^6^ iterations with a sampling every 500 iterations. Consensus tree and model parameter were determined after removing the 25% first iteration as burning. Convergence was evaluated for each parameter using ESS (Estimated Sample Size) with at least 50 and a PSRF (Potential Scale Reduction Factor) close to one. Similarly, two independent Markov chain Monte Carlo analysis and convergence was checked using average convergence of split less than 0.05. The tree was visualized midpoint rooted using iTOL v.6^40^. For strains isolated from animals and humans demonstrating extensive genomic similarity, as resulting from the Bayesian phylogenetic analysis, SNPs occurrence among these isolates was further investigated by snp-dists v.3.0 (https://github.com/tseemann/snp-dists).

Comparison of amount of resistance genes between clades was estimated by Wilcoxon-Mann Whitney test on BiostaTGV (https://biostatgv.sentiweb.fr/).

### Characterization of the genetic background of resistance genes

Plasmids and genetic elements carrying ARGs were analyzed by blastn and Easyfig 2.2.5_win for the 10 isolates sequenced by Oxford Nanopore technology and 8 isolates from NCBI repository having full-assembled genomes (Supplementary Table S2)^41,42^. Plasmids transferability was tested in vitro^43^ using rifampicin resistant *A. baumannii* strain BM4547 as recipient^44^. In plasmids, *rep* genes were characterized according to the Lam et al*.* scheme^45^.

## Results

### Features of animal and human isolates of this study

On average, sequencing of isolates of this study generated 2.36 M of reads per genome, with an estimated genome size of 4.2 M bases, with a coverage depth of 204X (Table S1).

Animal isolates included in this study (*n* = 33) were found mostly in urine (*n* = 14), followed by infected wounds (*n* = 8), respiratory samples (*n* = 4), eye swab (*n* = 3), and sporadically from body fluid (*n* = 1) and lymphatic ganglion (*n* = 1). Sampled animals came from 19 different departments located in seven regions of France (Table S1). Human isolates were found mostly during screening (rectal swabs) at hospital admission (*n* = 7), from urine (*n* = 3), from blood (*n* = 1) and infected wound (*n* = 1). The patients were resident in ten different departments (Table S1) from seven regions of France.

### Phylogenetic analysis of the ST25 *A. baumannii* lineage

From a first Bayesian phylogenetic analysis on 162 ST25 genomes and 24 ST25-SLV, available from the NCBI repository, on 2,386 core genes, only a part was retained for the final analysis. In particular, from each group of closely related genomes (< 5 different alleles), only one genome was included in the final phylogenetic analysis resulting in a set of 148 genomes (104 from the NCBI repository, and 44 from our collection). A total of 1,883 core genes were retained after exclusion of chromosomal genes presenting at least one variation, and those exhibiting potential recombination events (i.e. > 6 SNPs per 100-bp) presenting less than six SNPs per 100 bp, thus excluding potential frequent recombination.

**Figure 1:**
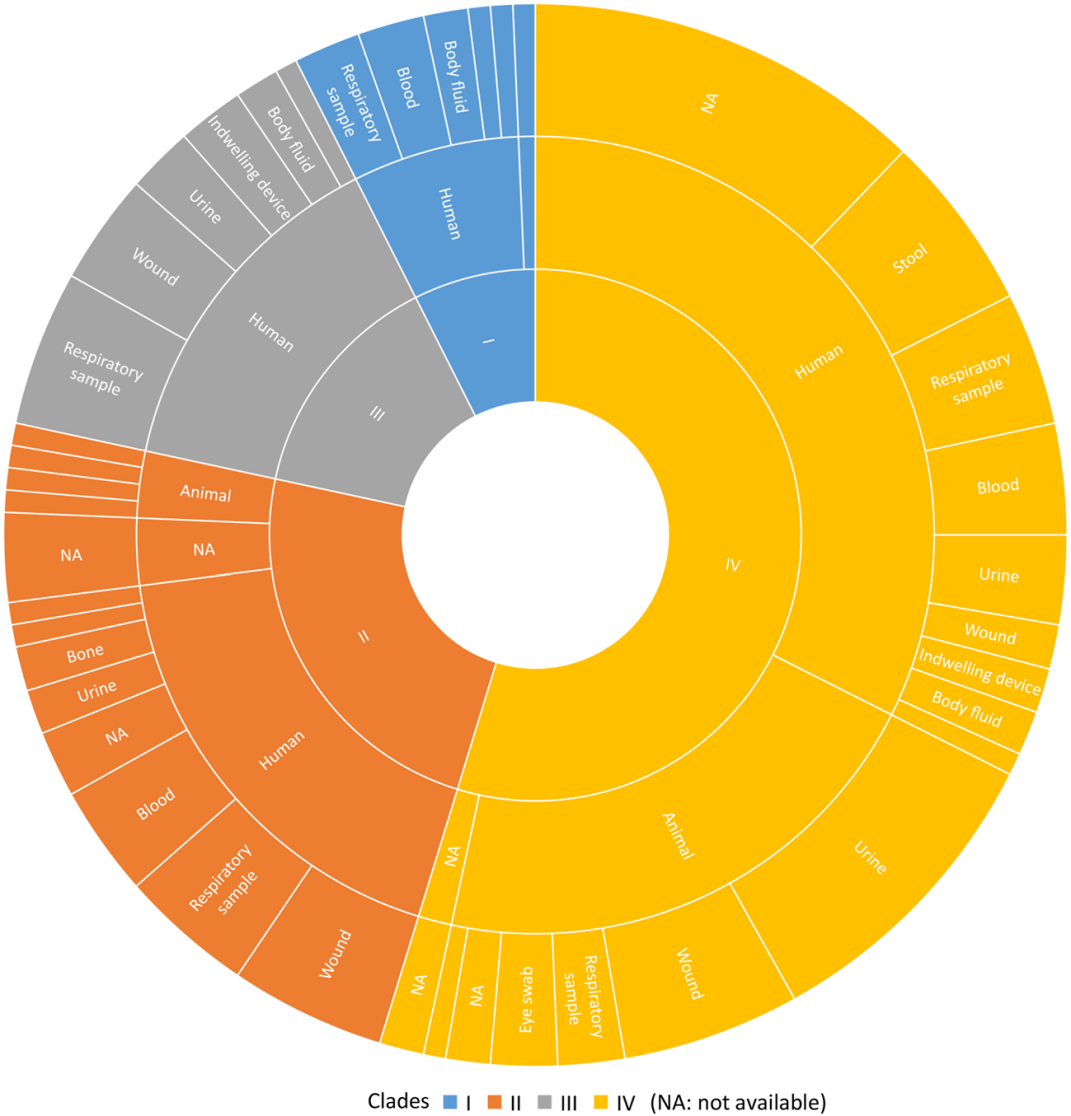
Host and biological sources of 148 genomes clustering in four (I, II, III, IV) clades.

**Figure 2:**
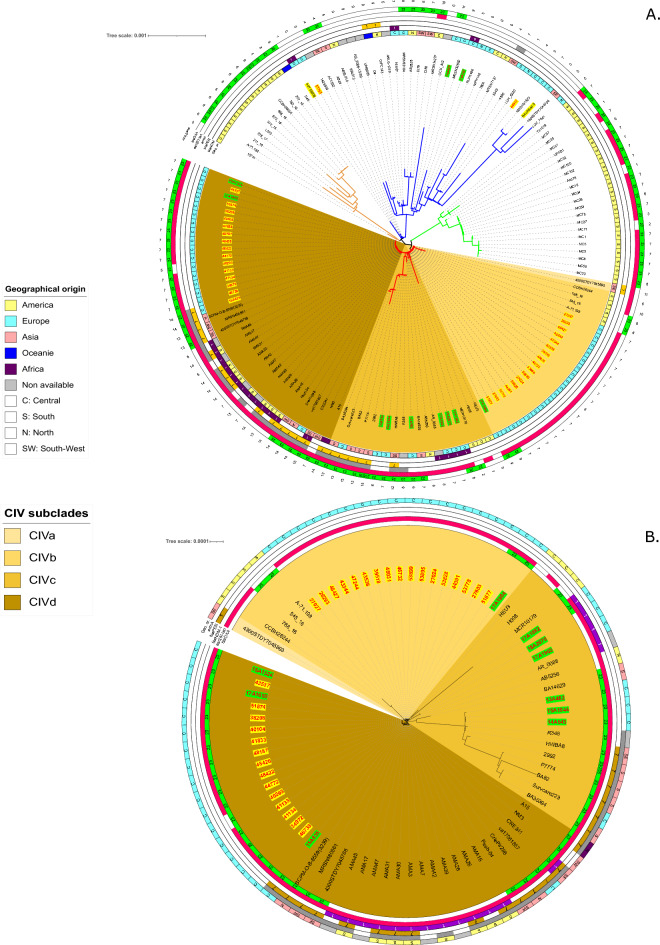
(**A)** Schematic representation of the phylogenetic relatedness (MrBayes) of 148 ST25 *Acinetobacter baumannii* genomes using iTOLv6. The tree was midpoint rooted and branch support was > 99%. These genomes belong to the 44 strains sequenced for the present study (red labels), and 104 other strains selected from the NCBI repository. Isolates recovered from animals are indicated by yellow shadowed labels, whereas those from humans of this study by light green. Colors of branches highlight four clades (CI: light orange; CII: blue; CIII: light green; CIV: red). (**B)** Zoom on the CIV clade.

The 148 ST25 *A*. *baumannii* genomes were clustered into four clades (CI, CII, CIII, CIV), that differed in size, geographical distribution, and host (Figs. 1 and 2A). Clade CI was composed of eleven isolates found exclusively in human patients from South America, between 2013 and 2019 (Fig. 1 and Supplementary Table S1). Similarly, clade CIII contained 22 isolates involved in human infections occurring in South America, between 2011 and 2016. The 35 strains that make up clade CII were collected from four continents and dated between 1985 and 2018. In this group, four isolates were recovered from animals, of which two collected in 2018, one from a dog in Germany (IHIT38008) and the other from a cat in France (53765), were closely related (31 SNPs between the two genomes). Our core genome analysis also showed that, to a less extent than the similarity reported above, strain MRSN31523 involved in a human bone infection in 2004 was phylogenetically close to a strain (BAuABod-3) isolated in 2015 from a turkey (145 SNPs). Although the two bacteria have been found in Germany, no clear epidemiological link could be established between them.

Besides being the clade gathering the most recent strains (*n* = 80), clade IV was the most heterogeneous group of ST25 *A. baumannii* regarding host origins (Fig. 1). Both animal- (*n* = 31/33 of our collection) and human-associated isolates (*n* = 47/106) clustered in this clade, with two additional strains being of unknown origin. Almost half of bacteria (*n* = 43) were collected in Central Europe, while the remaining 37 were from Asia, Central and South America, and North Africa (Fig. 2A). Four major subclades, from CIVa to CIVd, could be distinguished, with the CIVa subgroup represented only by a single strain (Fig. 2B). The 22 CIVb strains were isolated between 2010 and 2020 in Brazil (*n* = 4) and France (*n* = 18). Of these latter, 17 were found in animals (Supplementary Table S1). Interestingly, genome comparisons highlighted a relatedness between a strain (51877) responsible for a dog conjunctivitis in 2018, and a strain (17A1904) collected in 2017 from a patient’s wound (58 SNPs). Both were found in the South of France, but in two different departments. Subclade CIVc contained only human isolates from Europe and Asia and were more diverse compared to those included in subclade CIVb and CIVd (Fig. 2B).

Subclade CIVd turned out to contain genotypically close strains of *A.* *baumannii* ST25 from various geographical areas such as Asia, South America, and Europe. Fourteen of the 38 strains (37%) were from dogs and cats. All the European isolates originated from France, in particular the Paris area, but differed with respect to their hosts. Most of French human CIVd isolates were of fecal origin and were considered as a cause of colonization rather than infection. Extensive genomic similarities were revealed between two strains, 18A2070 and 17A1903 (21 SNPs), isolated from two patients hospitalized in two different towns in the Paris area, as well as between three isolates collected during 2013–2015 from patients residing in three different French towns (13A462 differed for 74 SNPs from 15A1044 and 14A543, whereas 14A543 and 15A1044 differed for only 18 SNPs). In addition, extensive genomic similarity concerned isolates from animals and humans (16A1524 from human stool and 43537 from cat urine (22 SNPs); 17A1650 from human urine and 51874 from a dog’s wound (24 SNPs); 15A835 from human stool and 46738 from a dog’s wound differing for 103 SNPs).

### Acquired resistome of the ST25 *A*. *baumannii* lineage

**Figure 3:**
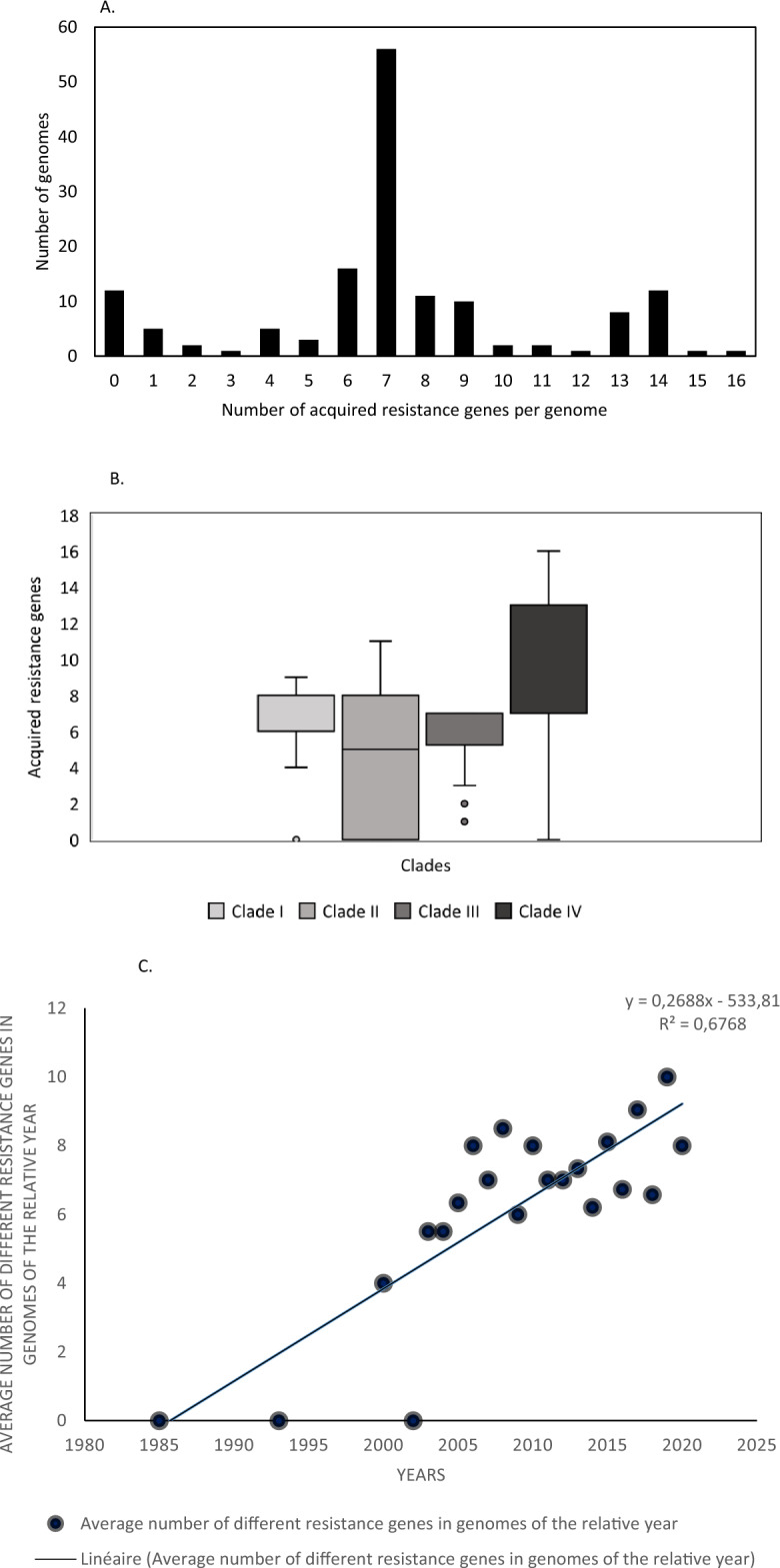
(**A)** Distribution of acquired antibiotic resistance genes among 148 *Acinetobacter baumannii* strains belonging to ST25 lineage, according to ResFinder 4.1. The modal number of ARGs was seven. (**B)** Acquired antibiotic resistance genes content of the four clades of ST25 lineage. Clade CIV harbored more antibiotic resistance genes than the other clades (*p* < 0.01, after Wilcoxon-Mann Whitney test; number of genomes per clade: CI: 11; CII: 35; CIII: 22; CIV: 80). (**C)** Scatter plot of the average number of acquired antibiotic resistance genes content of ST25 *Acinetobacter baumannii* genomes relative to time.

A total of 52 ARGs were identified in the 148 genomes examined (Supplementary Table S1), of which 48 were acquired horizontally (Supplementary Figure S1). The number of each different ARG per genome varied from 0 to 16 with a modal value of 7 (Fig. 3A). While genomes of clade CI, CII and CIII did not show significant differences in the amount of carried ARGs, genomes of clade CIV harbored more transmissible ARGs than the other clades (Wilcoxon-Mann Whitney test, *p* < 0.01) (Fig. 3B). The overall average of different ARGs of all genomes of the same year has been increasing during time (Fig. 3C), although with a R^2^ value of the linear regression close to 0.7, suggesting variability in this increase. The number of genomes available before 2005 differed considerably compared to the period 2005–2020, probably decreasing the statistical power of the model.

**Figure 4:**
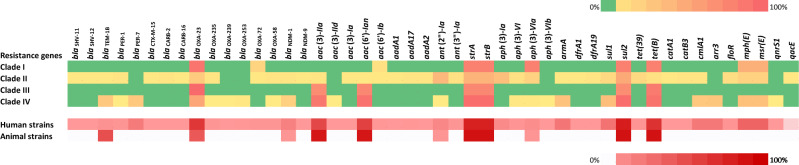
Heatmap representation of the occurrence of acquired antibiotic resistance genes in 148 ST25 *Acinetobacter baumannii* genomes according to phylogenetic grouping (number of isolates in each clade CI, n = 11; CII, n = 35; CIII, n = 22; CIV, n = 80) and host origin of the isolates.

Among the most widespread ARGs were those determining resistance to sulfonamides (*sul2* present in 81.8% strains), aminoglycosides (*strA* coexisting with *strB* in 79.7% strains), and tetracyclines (*tet*(*B*) in 70.9%) (Fig. 4)). Carbapenemase-encoding genes were also prevalent in the ST25 lineage, especially *bla*_OXA-23_ (56.8%), followed by *bla*_NDM-1_ (12.3%). Of note, 1 out of the 19 NDM-1 producing strains (5.3%) originated from an animal, a dog sampled in Germany^23^ while 17.9% (*n* = 15/84) of OXA-23 positive strains were of animal origin (6 from dogs, and 9 from cats). The prevalence of these two carbapenemase-encoding genes differed in animal and human strains. In particular, *bla*_OXA-23_ was present in 42.9% (*n* = 15/35) and 64.2% (*n* = 68/106) of animal and human strains, respectively, whereas *bla*_NDM-1_ was more represented in human strains (16.0%, *n* = 17/106) than in animal strains (2.9%, *n* = 1/35). Two human strains harbored the two genes simultaneously. Extended-spectrum β-lactamases (ESBLs) were more sporadic and exclusively produced by human strains, with CTX-M-15, SHV-11, and SHV-12 being harbored by single isolates, respectively and PER-1 by 3/106 isolates (2.8%). Interestingly, ESBL PER-7 was identified in 21/106 ST25 strains (19.8%), all belonging to the CIV clade, and containing the pan-aminoglycoside resistance gene *armA*. In contrast to other β-lactamase genes, those encoding penicillinase *bla*_TEM-1B_ were more predominant in animals (17/35; 48.6%) than in humans (5/106; 4.7%).

As noted above, distribution of acquired ARGs was uneven among the various ST25 clades. Almost all the CIVc and CIVd strains were either OXA-23 (37/57; 64.9%), NDM-1 (15/57; 26.3%) or OXA-23/NDM-1 (2/57; 3.5%) positive, NDM-1 being co-produced with PER-7 and ArmA in seven bacteria. Though OXA-23-carrying rates varied in the other clades or subclades including CI (10/11; 90.1%), CII (9/35; 25.7%), CIII (22/22; 100%), CIVa (0/1, 0%), and CIVb (4/22; 18.2%), our analysis revealed that only two and one CII strains had acquired the genes encoding NDM-1 and ArmA, respectively. Similarly, aminoglycoside resistance genes *aac*(6’)-*Ian* (93/148; 62.8%) and *aac*(3)-*IIa* (89/148; 60.1%) were almost exclusively identified in clades CIII (17/93 and 16/89) and CIV (75/93 and 71/89) (Fig. 4, Table S1).

### Genetic elements harboring *bla*_OXA-23_ gene and their integration sites

Because of the high prevalence of carbapenemase OXA-23 among animal and human ST25 *A. baumannii*, we looked at the genetic environment of this gene in strains for which full-assembled genomes were available, from our study (38208, 51877, 13A462, 14A453, 15A1044, and), and from the NCBI database (P7774, HWBA8, UPAB1, CriePir298, 2992).

Isolate 38208 from clade CIVd harbored a duplicate *bla*_OXA-23_ gene on two copies of transposon Tn*2006*^46^. One copy of Tn*2006* was inserted into the *tssi* gene, homologous to the locus D1G37_06470 (CP032215.1) encoding a component of T6SS, and was flanked by 9 bp direct repeat (DR) sequence 5′-AAGCTGACT-3′. This integration site is uncommon for Tn*2006*^47^. To understand if gene *tssi* was a common integration site for Tn*2006* in ST25 lineage, we analyzed the integrity of *tssi* gene in all 148 genomes. Although Tn*2006* was not observed in this locus in other ST25 isolates, most likely because of assembly level, the *tssi* gene was found split into two contigs in 17 out of the 38 isolates of sub-clades CIVd (Supplementary Table S1). The other copy of Tn*2006*, bracketed by 9 bp DR sequence (5′-ATTCGCGGG-3′), was located in an AbaR4-type resistance island sharing 99% nucleotide identity with the previously characterized AbaR4 (JN107991.2). The *bla*_OXA-23_ gene was located on an AbaR4-type element also in 13A462, 14A543, 15A1044, HWBA8, P7774, CriePir298, and 2992. In all these strains and 38208, the AbaR4-type, flanked by 5 bp DR (5’-GCGGT-3’), was inserted into the *comM* gene at 841 bp downstream from the initiation codon. The *comM* gene is the most common locus for AbaR integration in *A. baumannii*^48^. Like *tssi* gene, we analyzed the integrity of the *comM* gene in all 148 genomes. Although AbaR4 or other elements were not observed in this locus in other ST25 isolates, splitting of the *comM* locus into two contigs occurred in one isolate out of 11 of clade CI and in 49% of CII clade isolates (*n* = 17/35). The gene *comM* was also split in isolates of the CIV clade, in 58% (*n* = 11/19) of isolates constituting the sub-clade CIVc, and in 66% (*n* = 25/38) of CIVd isolates. Overall, 36% (*n* = 52/148) of the ST25 isolates had a disrupted *comM* locus, but none of those belonged to the CIII or CIVa/b clades (Supplementary Table S1).

**Figure 5:**
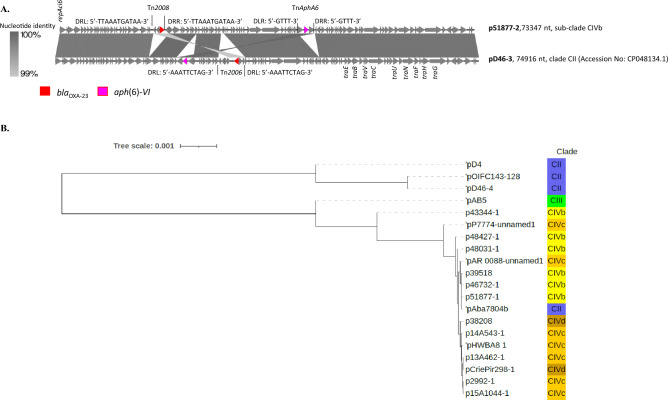
(**A)** Representation of blastn alignment of plasmids harboring a *bla*_OXA-23_ and *aph*(6)-*VI* genes and their transposons. (**B)** Tree representation (Neighbor join) of blastn alignment of large plasmids (> 132-k nucleotides) found in isolates clustering in different clades. Plasmid pD46-4 was used as reference. Unit of tree scale was base substitutions per site (iTOL v.6).

In clade CIII isolate UPAB1, the *bla*_OXA-23_ gene was carried by a Tn*2008* element^49^ inserted between chromosomal loci D1G37_09730 and D1G37_09750 (CP032215.1) without interrupting them and surrounded by 9 bp DR (5′-TGAATTTTT-3′). Previously, Tn*2008* has been found in several chromosomal loci and on plasmids^50,51^. In CIVb isolate 51877, the *bla*_OXA-23_-bearing Tn*2008* transposon was localized on a plasmid, named p51877-2. This latter shared extensive similarity with pD46-3 of CII D46 isolate (Fig. 5A, Table S2), which instead carried a Tn*2006*^52^. A copy of Tn*AphA6* transposon^53^, bracketed by 5′-GTTT-3′ DR sequence and bearing the *aph*(3’)-*VI* gene, which confers resistance to amikacin^54^, was also present on these plasmids (Fig. 5A). Tn*AphA6* has been reported from isolates belonging to IC1^55^ and IC2^53^. Consistent with the presence of numerous transfer genes *tra* and the replicase *repAci6* gene on both plasmids, we observed the transfer p51877-2 to a recipient *A*. *baumannii* strain in vitro (data not shown). Other plasmids possessing RepAci6 (RP-T1) have been demonstrated to transfer in vitro, like pACICU2 (from isolate *A*. *baumannii* ACICU, ST2) which also harbors gene *bla*_OXA-23_ and a Tn*AphA6*^53^.

Genetic elements could be analyzed in a small proportion of ST25 genomes. However, this analysis highlighted the diversity of elements carrying *bla*_OXA-23_ in ST25 lineage suggesting the occurrence of different and independent acquisition of this gene in the different clades.

### Genetic elements and plasmids harboring other resistance genes

The resistance genes *sul2*, *strB, strA* and *tet*(B), frequently identified in IC2 strains^48^, were widespread in clone ST25, with more than 70% of ST25 isolates carrying them. In clade CII (D4, OIFC143, 7804) and CIII (UPAB1) these genes were located on a Tn*6172*-type transposon. In isolates belonging to clade CIV, all these ARGs were detected on elements derived from transposon Tn*6172*^56^ that was adjacent to a Tn*6022*-like element. Overall this structure resembled an AbGRI-1 variant^56^ (Supplementary Figure S1A, Table S2). AbGRI-1 is commonly detected in the chromosome of strains belonging to IC2^48^ whereas in the ST25 isolates of this study, AbGRI-1 variant was located on large plasmids without a previously characterize *rep* gene, ranging in size from 145,709 to 232,318-bp, and for which we did not observe in vitro transfer. These plasmids harbored other resistance genes, such as *bla*_TEM-1B_ in animal isolates (Supplementary Table S2).

Clade CII D46 isolate, three of clade CIVc and CriePir298 of clade CIVd harbored large plasmids that were positive for R3-T60 Rep-type^45^. However, this *rep* type is located on a resistance island and does not appear to contribute to the replication process of the plasmid^57^. These plasmids, harbored several resistance genes (including *bla*_PER-7_ and *armA*) on complex genetic regions characterized in previous studies^57–60^ (Supplementary Figure S1B and C, Table S2). Furthermore, all large plasmids of CIV clade isolates carried the *aac*(3)-*IIa* and *aac*(6’)-*Ian* aminoglycoside resistance genes, borne by a 8,138 bp long transposon containing two copies of IS*6*, and flanked by IS*91* (Supplementary Figure S1D, Table S2). Similarities searches in the NCBI database revealed that this transposon was present on plasmids previously reported in *Klebsiella pneumoniae* (e.g., plasmid pKp 1050–4 in strain CP023420.1), and *Serratia marcescens* (plasmid p1163 in strain AP01411.1).

Besides antibiotic resistance genes, these large plasmids carried genes encoding regulators of chromosomal genes involved in virulence ^61,62^, a factor that probably contributed to their maintenance in the cell (Supplementary Table S3). Alignment of large plasmids demonstrated that similarity among them mostly reflected clade grouping of the chromosomes (Fig. 5B). Plasmids carried large conserved regions (data not shown), but strains belonging to a specific clade harbored similar genetic elements potentially explaining the observed divergence.

## Discussion

The ST25 *A*. *baumannii* lineage has diversified in four clades (CI–CIV) with different geographical and temporal expansion. These data build upon those from Sahl et al*.*, who demonstrated a degree of divergence in the genomes constituting the ST25 lineage^63^. Here, CI and CIII clades each appear to be restricted to two countries in South America, Brazil and Bolivia, respectively, where ST25 *A*. *baumannii* is endemic^4,64^. On the contrary, CII and CIV have expanded globally. In particular, the CIV clade, which includes most of the isolates of this study, contains most of the multidrug resistant and recent isolates, suggesting its continuous spread and evolution. Several of them were isolated from animals, and some carried an acquired carbapenemase-encoding gene. Carbapenem-resistant strains were found in dogs and cats in our study, whereas ST25 *A*. *baumannii* found in horses were susceptible to this class of antibiotics. More generally, *A*. *baumannii* strains have been isolated from adult horses and foals in numerous studies^20,65–68^, but carbapenem-resistant *A*. *baumannii* from horses are rare^69^. Carbapenem-based therapies are not approved for use in animals in Europe, except in exceptional cases^70^. Other β-lactams are commonly used in animals, such as amoxicillin and amoxicillin combined with clavulanic acid^70^. While exposure of all animal hosts to third and fourth generation cephalosporins has enormously decreased (− 94.3%) during 2011–2020 in France, exposure of dogs and cats to amoxicillin and amoxicillin associated to clavulanic acid has not decreased significantly (− 1.6%)^71^. Although these molecules are a substrate for OXA-23^68^, it is likely that the selective antibiotic pressure is not the only driver of the spread of this resistance mechanism in dogs and cats. For instance, hospitalization in veterinary clinics has been recognized as a risk factor for colonization of pets with carbapenem-resistant isolates ^29,72^; this exemplified on our study by the occurrence of three isolates (40293, 41133, and 41134) that were collected from three animals attending the same veterinary clinic^30^. Other isolates analyzed in this study were found in animals that were in poor health and required frequent hospitalizations. However, according to the diagnostic veterinary records, some animals were attending the veterinary clinic for the first time and became infected with carbapenem-resistant isolates less than 48 h after admission, suggesting that colonization occurred first in the community. Hérivaux et al. screened dogs and cats (*n* = 150) attending a preventive veterinary clinic. Four dogs (2.7%) with no history of antibiotic exposure or previous hospitalization were colonized with *A*. *baumannii* isolates and two were ST25 OXA-23 producers, demonstrating that pets can be a reservoir of carbapenem-resistant *A*. *baumannii* in the community^31^. The source of carbapenem-resistant *A*. *baumannii* causing pets’ colonization remains to be elucidated. Our study highlighted the occurrence of phylogenetically closely related carbapenem-resistant isolates (differing by less than 30 SNPs) collected from pets (dogs and cats) and colonizing humans, screened at hospital admission. The epidemiological data (date of collection, residence of colonized humans and pets) exclude the possibility of a direct transmission of these isolates, but overall, shuffling of carbapenem-resistant *A*. *baumannii* clones between humans and pets cannot be excluded. In fact, a ST1386 *A*. *baumannii* strain that caused otitis in a dog was found colonizing its owner’s nose, providing further warning of the ability of *A*. *baumannii* to spread between various hosts^73^.

Although data on history travel of pets carrying ST25 *A*. *baumannii* were not available, thus import of the isolates cannot be completely excluded, it seems that ST25 is the most prevalent clone among *A*. *baumannii* circulating in diseased French animals. Recently, Jacobmeyer et al*.* analyzed 29 carbapenemase-producing *A*. *baumannii* isolates collected from animals attending a veterinary clinic^74^. Nine of these isolates were from seven veterinary clinics from France and five of them belonged to ST25. Carbapenemase-producing ST25 isolates were also found among German and Italian pets, but less frequently (3/16 and 1/3, respectively)^74^. These results further corroborated the general importance of ST25 circulation among companion animals, particularly in France. This epidemiological situation is surprising, given that most of human carbapenem-resistant isolates belong to ST2^3^. When we looked at the clonal relationship between ST2 and ST25, an eburst analysis on the available 2262 sequence types (data not shown) suggested that ST2, ST25, and their single locus variants form two distinct clonal complexes. Epidemiological data in humans derive from the hospital sector and colonization of human population by carbapenem-resistant *A. baumannii* outside nosocomial settings remains largely unknown.

Resistance genes widely distributed in IC2 isolates (*sul2*, *strA*, *strB,* and *tet*(B)) and related genetic elements were also found in ST25 *A*. *baumannii* isolates. In fact, the *strA*, *strB*, *sul2* and *tet*(B) genes were frequently found in regions similar to the AbRGI-1 genetic element, especially to the variant reported in ST2 isolates who carry AbRGI-1 mostly into the chromosome^56^. However, this genetic element was mainly located on large plasmids in the ST25 isolates analyzed in this study. In addition, these plasmids carried the *aac*(3)-*IIa* and *aac*(6’)-*Ian* genes, which are located on a transposon widely distributed among Enterobacterales. Plasmids from clade CIV isolates carried genes predicting a ESBL phenotype (*bla*_PER-7_) and pan-aminoglycosides resistance (*armA*) in addition to resistance genes for old antibiotics (*sul2*, *tet*(B), *strA* and *strB*). Although the in vitro transfer of these large plasmids to recipient cells did not occur, their presence in animal isolates is of great concern, impeding the use of antibiotics commonly prescribed for treating animals. Besides treatment failure, their presence could favor the development of further resistances through co-selection processes. In parallel with the lack of in vitro transferability, the genetic relatedness of plasmids from isolates of different clades reflected that of genome clustering, with plasmids from clade CIV being more closely related to each other than to those of isolates of clade CII and CIII, suggesting that plasmids co-evolved with the chromosome of their host strains. In fact, large plasmids of ST25 genomes contain genes homologous to loci previously characterized and shown to encode regulators of chromosomal-located genes involved in virulence. These regulators include those of TetR type, which repress chromosomal genes encoding the T6SS. Di Venanzio et al*.* observed that the repression of T6SS could favor the successful transfer of plasmids to recipient hosts without killing them, thus contributing to antibiotic resistance genes dissemination^75^. Intriguingly, in 38208 strain, the *tssi* gene, predicted to encode a VgrG4 protein sharing similarities with the terminal part of the T6SS pilus, was disrupted by the insertion of Tn*2006* transposon. Similarly to the repression of the T6SS, the *tssi* disruption could affect the ability of the isolates to kill other bacteria by preventing the production of an effective T6SS and favor conjugation^76^. This physiological advantage could justify the duplication of Tn*2006*. In 38208 strain, in fact, another copy of Tn*2006* was present in an AbaR4, inserted into the *comM* gene, which has been shown to be involved in natural transformation^77^. However, if inactivation of T6SS seems to favor conjugation, in this study, in vitro assays did not detect the transfer of the large plasmids present in the strain. The disruption of the *tssi* gene was observed only in part of the CIVd isolates. Godeux et al. provided experimental evidence for the role of transformation as a key mechanism for acquisition of resistance island in *Acinetobacter* spp., that could then clonally disseminate integrated into the *comM* locus^47^. This is the most common locus of resistance island integration in several *A*. *baumannii* clones and probably also in ST25 clone, where this locus was disrupted in 36% of genomes.

## Conclusion

The ST25 *A*. *baumannii* lineage continues to spread. Within this lineage, the here-defined clade CIV is of particular concern, as it includes genomes from recently reported human and animal isolates that have developed multidrug resistance. The CIV clade of the ST25 lineage might achieve a similar success to that of IC2, thus its propagation among humans and animals needs to be monitored.

Companion animals in France, especially dogs and cats may nourish the circulation of carbapenem-resistant ST25 strains with potential for human colonization. Multidrug resistant ST25 strains may act as donors of resistance genes and their genetic element for susceptible clones.

### Supplementary Information

Supplementary Table S1.

Supplementary Table S2.

Supplementary Table S3.

Supplementary Figure S1.

## Data Availability

Genome sequences of isolates sequenced for this study are available from PRJNA838428 and PRJNA766794 (https://www.ncbi.nlm.nih.gov/bioproject).
