# Supplementary material for: Multiple host colonization and differential expansion of multidrug-resistant ST25-Acinetobacter baumannii clades
Source: Sci Rep. 2023 Dec 9;13:21854. doi: 10.1038/s41598-023-49268-x (PMC10710421; doi:10.1038/s41598-023-49268-x)
Supplement: Supplementary file 2 — Supplementary Table S2. [file 41598_2023_49268_MOESM2_ESM.docx]

|  | **Table S2. Plasmid content of ST25 *Acinetobacter baumannii* isolates for which full assembled genome was available.** | | | | | | |
| --- | --- | --- | --- | --- | --- | --- | --- |
| Isolate | | Plasmid name | Plasmid Accession No | Clade | Plasmid size (nt) | Rep type | Antibiotic resistance genes (genetic element) |
| D46 | | pD46-4 | CP048135.1 | II | 208004 | - | *tet*(B), *sul2*, *strA*, *strB*, *msrE*, *mphE*  (Tn*6183*)^1^ |
|  | | pD46-3 | CP048134.1 |  | 74916 | RP-T1 | *bla*_OXA-23_ (Tn*2006*)^2^, *aph*(3’)-*VIa* (Tn*AphA6*)^2^ |
|  | | pD46-2 | CP048133.1 |  | 8731 | R3-T1 | None |
|  | | pD46-1(pRAY) | CP048132.1 |  | 6078 | - | *ant*(2’’)-*Ia* (*aadB*)^3^ |
| D4 | | pD4 | CP048851.1 | II | 132632 | - | *sul2*, *strA*, *strB* (Tn*6172*)^4^ |
|  | | pD4-1 | CP048850.1 |  | 2277 | R3-T15 | None |
| OIFC143 | | OIFC143-128 | AFDL01000008.1 | II | 127633 | - | *sul2*, *strA*, *strB* (Tn*6172*)^4^ |
|  | | OIFC143-70 | AFDL01000006.1 |  | 69518 | RP-T1 | None |
|  | | OIFC143-6.2 | AFDL01000007.1 |  | 6241 | R3-T5 | None |
|  | | OIFC143-2.3 | AFDL01000005.1 |  | 2277 | R3-T15 | None |
| 7804 | | pAba7804b | CP022285.1 | II | 170420 | - | *sul2*, *strA*, *strB* (Tn*6172*-like)*^5^* |
|  | | pAba7804a | CP022284.1 |  | 12381 | R3-T17 | *bla*_OXA-58_ |
| UPAB1 | | pAB5 | CP032216.1 | III | 100163 | - | *sul2*, *strA*, *strB* (Tn*6172*) |
|  | | pUPAB1-unn1 | CP032217.1 |  | 80061 | R3-T3 | None |
|  | | pUPAB1-unn2 | CP032220.1 |  | 16743 | - | None |
| **48427** | | **p48427-1** |  | **IVb** | **207299** | **-** | ***tet*(B), *sul2*, *strA*, *strB* (AbGRI1-like), *bla*_TEM-1B_**  ***acc*(3)-*IIa*, *acc*(6’)-*Ian*** |
|  | | **p48427-2** |  |  | **6291** | **R3-T15** | **None** |
|  | | **p48427-3** |  |  | **1262** | **-** | **None** |
| **43344** | | **p43344** |  | **IVb** | **232318** | **-** | ***tet*(B), *sul2*, *strA*, *strB* (AbGRI1-like), *bla*_TEM-1B_** |
|  | |  |  |  |  |  | ***acc*(3)-*IIa*, *acc*(6’)-*Ian*** |
| **39518** | | **p39518** |  | **IVb** | **172572** | **-** | ***tet*(B), *sul2*, *strA*, *strB* (AbGRI1-like), *bla*_TEM-1B_** |
|  | |  |  |  |  |  | ***acc*(3)-*IIa*, *acc*(6’)-*Ian*** |
| **48031** | | **p48031-1** |  | **IVb** | **211829** | **-** | ***tet*(B), *sul2*, *strA*, *strB* (AbGRI1-like), *bla*_TEM-1B_** |
|  | |  |  |  |  |  | ***acc*(3)-*IIa*, *acc*(6’)-*Ian*** |
|  | | **p48031-2** |  |  | **7780** | **R3-T12** | **None** |
|  | | **p48031-3** |  |  | **6078** | **-** | ***ant*(2’’)-*Ia* (partial)** |
| **46732** | | **p46732-1** |  | **IVb** | **211829** | **-** | ***tet*(B), *sul2*, *strA*, *strB* (AbGRI1-like), *bla*_TEM-1B_,** |
|  | |  |  |  |  |  | ***acc*(3)-*IIa*, *acc*(6’)-*Ian*** |
|  | | **p46732-2** |  |  | **10979** | **R3-T12** | **None** |
|  | | **p46732-3** |  |  | **6078** | **-** | ***ant*(2’’)-*Ia*** |
| **51877** | | **p51877-1** |  | **IVb** | **230883** | **-** | ***tet*(B), *sul2*, *strA*, *strB* (AbGRI1-like), *bla*_TEM-1B_** |
|  | |  |  |  |  |  | ***acc*(3)-*IIa*, *acc*(6’)-*Ian*** |
|  | | **p51877-2** |  |  | **73347** | **RP-T1** | ***bla*_OXA-23_ (Tn*2008*), *aph*(3’)-*VIa* (Tn*AphA6***) |
|  | | **p51877-3** |  |  | **6326** | **-** | **None** |
| AR_0088 | | pAR_0088_1 | CP027531.1 | IVc | 146698 | - | *tet*(B), *sul2*, *strA*, *strB* (AbGRI1-like)*^6^* |
|  | |  |  |  |  |  | *acc*(3)-*IIa*, *acc*(6’)-*Ian* |
|  | | pAR_0088_2 | CP027532.1 |  | 41087 | - | *bla*_NDM-1_, *aph*(3’)-*VIa* (Tn*7382*-like)^7^ |
| **13A462** | | **p13A462-1** |  | **IVc** | **164402** | **-** | ***sul2*, *strA*, *strB* (AbGRI1-like)** |
|  | |  |  |  |  |  | ***acc*(3)-*IIa*, *acc*(6’)-*Ian*** |
|  | | **p13A462-2** |  |  | **2292** | **-** | **None** |
| **15A1044** | | **p15A1044-1** |  | **IVc** | **184002** | **-** | ***sul2*, *strA*, *strB* (AbGRI1-like)** |
|  | |  |  |  |  |  | ***acc*(3)-*IIa*, *acc*(6’)-*Ian*** |
| **14A543** | | **p14A543-1** |  | **IVc** | **166840** | **-** | ***sul2*, *strA*, *strB* (AbGRI1-like)** |
|  | |  |  |  |  |  | ***acc*(3)-*IIa*, *acc*(6’)-*Ian*** |
|  | | **p14A543-2** |  |  | **3548** | **-** | **None** |
| 2992 | | pUnnamed 1 | VYJD01000012.1 | IVc | 198655 | - | *tet*(B), *sul2, bla_PER-7_, armA, arr-2, cmlA1, msrE*, *mphE, sul1* (RI-PER-7)^6^ |
|  | |  |  |  |  |  | *acc*(3)-*IIa*, *acc*(6’)-*Ian* |
| CriePir298 | | pUnnamed1 | JAEPWB010000002.1 | IVd | 183139 | - | *tet*(B), *sul2, bla_PER-7_, armA, arr-2, cmlA1, ^8^msrE*, *mphE, sul1* (RI-PER-7)^6^ |
|  | |  |  |  |  |  | *acc(3)-IIa, acc(6’)-Ian* |
|  | | pUnnamed2 | JAEPWB010000003.1 |  | 7540 | R3-T1 | None |
| HWBA8 | | pHWBA8_1 | CP020596.1 | IVc | 195838 | - | *tet*(B), *sul2, bla_PER-7_, armA, arr-2, cmlA1, msrE*, *mphE, sul1* (RI-PER-7)^6^ |
|  | |  |  |  |  |  | *acc*(3)-*IIa*, *acc*(6’)-*Ian* |
| P7774 | | P7774-unn1 | CP040260.1 | IVc | 202283 | - | *tet*(B), *sul2, bla_PER-7_, armA, arr-2, cmlA1, msrE*, *mphE, sul1*(RI-PER-7)^6^ |
|  | |  |  |  |  |  | *acc*(6’)-*Ian* |
|  | | P7774-unn2 | CP040261.1 |  | 14880 | R3-T1 | None |
|  | | P7774-unn3 | CP040262.1 |  | 5464 | R3-T54 | None |
| **38208** | | **p38208** |  | **IVd** | **145709** | **-** | ***tet*(B), *sul2*, *strA*, *strB* (AbGRI1-like)** |
|  | |  |  |  |  |  | ***acc*(3)-*IIa*, *acc*(6’)-*Ian*** |

Note: -, no homology found with any of the previously characterized *rep* genes ^8^. Features of isolates sequenced in the current study are highlighted in bold.

1 Nigro, S. J. & Hall, R. M. A large plasmid, pD46-4, carrying a complex resistance region in an extensively antibiotic-resistant ST25 *Acinetobacter baumannii*. *J Antimicrob Chemother* **72**, 3496-3498, doi:10.1093/jac/dkx287 (2017).

2 Nigro, S. J., Holt, K. E., Pickard, D. & Hall, R. M. Carbapenem and amikacin resistance on a large conjugative *Acinetobacter baumannii* plasmid. *J Antimicrob Chemother* **70**, 1259-1261, doi:10.1093/jac/dku486 (2015).

3 Hamidian, M., Nigro, S. J. & Hall, R. M. Variants of the gentamicin and tobramycin resistance plasmid pRAY are widely distributed in *Acinetobacter*. *J Antimicrob Chemother* **67**, 2833-2836, doi:10.1093/jac/dks318 (2012).

4 Hamidian, M. & Hall, R. M. The resistance gene complement of D4, a multiply antibiotic-resistant ST25 *Acinetobacter baumannii* isolate, resides in two genomic islands and a plasmid. *J Antimicrob Chemother* **71**, 1730-1732, doi:10.1093/jac/dkw041 (2016).

5 Perez-Oseguera, A. *et al.* Complete Genome Sequence of a blaOXA-58-Producing *Acinetobacter baumannii* Strain Isolated from a Mexican Hospital. *Genome Announc* **5**, doi:10.1128/genomeA.00949-17 (2017).

6 Adams, M. D. *et al.* Distinct Mechanisms of Dissemination of NDM-1 Metallo-beta-Lactamase in *Acinetobacter* Species in Argentina. *Antimicrob Agents Chemother* **64**, doi:10.1128/AAC.00324-20 (2020).

7 Hamed, S. M., Hussein, A. F. A., Al-Agamy, M. H., Radwan, H. H. & Zafer, M. M. Tn*7382*, a novel composite transposon harboring *bla*_NDM-1_ and *aphA6* in *Acinetobacter baumannii*. *J Glob Antimicrob Resist* **30**, 414-417, doi:10.1016/j.jgar.2022.08.001 (2022).

8 Lam, M. M. C., Koong, J., Holt, K. E., Hall, R. M. & Hamidian, M. Detection and Typing of Plasmids in *Acinetobacter baumannii* Using *rep* Genes Encoding Replication Initiation Proteins. *Microbiol Spectr* **11**, e0247822, doi:10.1128/spectrum.02478-22 (2023).
