# Supplementary material for: Multiple host colonization and differential expansion of multidrug-resistant ST25-Acinetobacter baumannii clades
Source: Sci Rep. 2023 Dec 9;13:21854. doi: 10.1038/s41598-023-49268-x (PMC10710421; doi:10.1038/s41598-023-49268-x)
Supplement: Supplementary file 3 — Supplementary Table S3. [file 41598_2023_49268_MOESM3_ESM.docx]

| **Table S3. Presence and identity of loci encoding regulators of chromosomal genes involved in virulence in plasmids of isolates belonging to the ST25 *Acinetobacter baumannii* lineage.** | | | | | | |
| --- | --- | --- | --- | --- | --- | --- |
| Plasmid | Aminoacid Identity (%) | | | | | |
|  | H-NS protein (locus D1G37_RS18580) | TetR/AcrR regulator (locus D1G37_RS18620) | TetR/AcrR regulator (locus D1G37_RS18650) | TetR regulator (locus D1G37_RS18810) | Transcriptional regulator (locus D1G37_RS18815) | Metal/formaldehyde-sensitive transcriptional repressor (locus D1G37_RS18965) |
| pD46-4 | 99% (N17D) | 98% (N17D ; N37S ; F144I) | 97% (N71S ; A83D ; A94T ; C144S ; N178S) | 100% | 100% | 100% |
| pD46-3 | - | - | - | - | - | - |
| pD46-2 | - | - | - | - | - | - |
| pD46-1 | - | - | - | - | - | - |
| pD4 | 99% (N17D) | 98% (N17D ; N37S ; F144I) | 97% (N71S ; A83D ; A94T ; C144S ; N178S) | - | - | 100% |
| pD4-1 | - | - | - | - | - | - |
| OIFC143-128 | 99% (N17D) | 98% (N17D ; N37S ; F144I) | 97% (N71S ; A83D ; A94T ; C144S ; N178S) | - | - | 100% |
| OIFC143-70 | - | - | - | - | - | - |
| OIFC143-6.2 | - | - | - | - | - | - |
| OIFC143-2.3 | - | - | - | - | - | - |
| pAba7804b | 100% | 100% | 100% | -* | -* | 100% |
| pAba7804a | - | - | - | - | - | - |
| pAB5 | 100% | 100% | 100% | 100% | 100% | 100% |
| pUPAB1-unn1 | - | - | - | - | - | - |
| pUPAB1-unn2 | - | - | - | - | - | - |
| **p48427-1** | 100% | 100% | 100% | 100% | 100% | - |
| **p48427-2** | - | - | - | - | - | - |
| **p48427-3** | - | - | - | - | - | - |
| **p43344** | 100% | 100% | 100% | 100% | 100% | 100% |
| **p39518** | 100% | 100% | 100% | 100% | 100% | - |
| **p48031-1** | 100% | 100% | 100% | 100% | 100% | 100% |
| **p48031-2** | - | - | - | - | - | - |
| **p48031-3** | - | - | - | - | - | - |
| **p46732-1** | 100% | 100% | 100% | 100% | 100% | 100% |
| **p46732-2** | - | - | - | - | - | - |
| **p46732-3** | - | - | - | - | - | - |
| **p51877-1** | 100% | 100% | 100% | 100% | 100% | 100% |
| **p51877-2** | - | - | - | - | - | - |
| **p51877-3** | - | - | - | - | - | - |
| pAR_0088_1 | 100% | 100% | 100% | 100% | 100% | - |
| pAR_0088_2 | - | - | - | - | - | - |
| **p13A462-1** | 100% | 100% | 100% | - | - | 100% |
| **p13A462-2** | - | - | - | - | - | - |
| **p15A1044** | 100% | 100% | 100% | 100% | 100% | 100% |
| **p14A543-1** | 100% | 100% | 100% | - | - | 100% |
| **p14A543-2** | - | - | - | - | - | - |
| p2992-1 | 99% | 100% | 100% | 99.8% | 100% | 100% |
| pCriePir298-1 | 100% | 100% | 100% | 99.8% | 100% | 100% |
| pCriePir298-1 | - | - | - | - | - | - |
| pHWBA8_1 | 100% | 100% | 100% | 100% | 100% | 100% |
| P7774-unn1 | 100% | 100% | 100% | 100% | 100% | 100% |
| P7774-unn2 | - | - | - | - | - | - |
| P7774-unn3 | - | - | - | - | - | - |
| **p38208** | 100% | 100% | 100% | 100% | 100% | - |

Note. -: absent; *presented two nucleotides deletion causing frameshift. In bold are highlighted plasmids sequenced for this study.
