# Supplementary material for: Multiple host colonization and differential expansion of multidrug-resistant ST25-Acinetobacter baumannii clades
Source: Sci Rep. 2023 Dec 9;13:21854. doi: 10.1038/s41598-023-49268-x (PMC10710421; doi:10.1038/s41598-023-49268-x)
Supplement: Supplementary file 4 — Supplementary Figure S1. [file 41598_2023_49268_MOESM4_ESM.pdf]

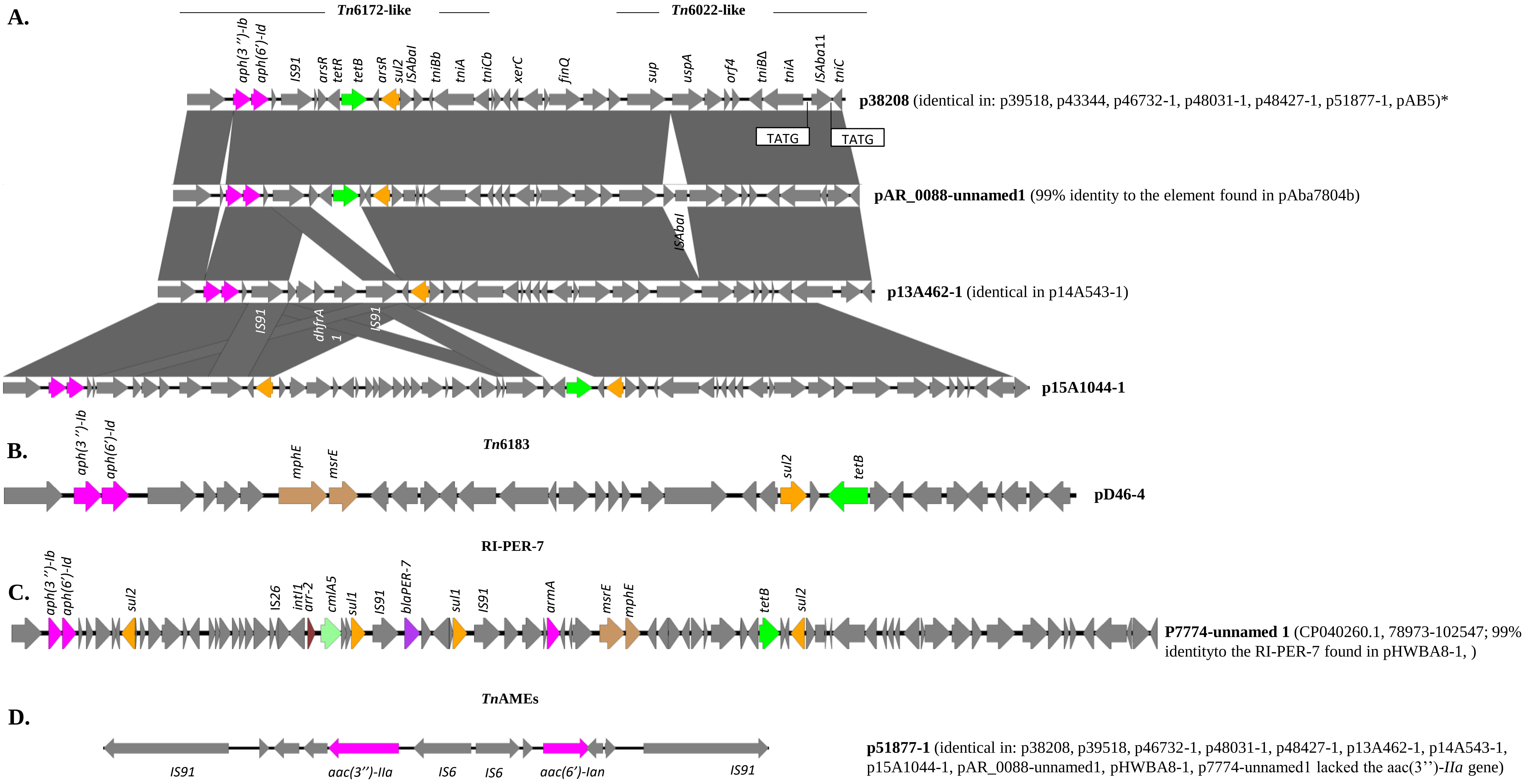

Supplementary Figure S1. Genetic elements harboring most common antibiotic resistance genes in ST25 full sequenced isolates. A. Representation (EasyFig_blastn v. 2.2.5) of the AbaGRI1 variant in isolates of this study and in genomes retrieved from NCBI.*: in isolates D4 and OIFC-143-128 the Tn*6022*-like element was missing. B. Representation of Tn*6183* with *msrE* and *mphE* genes in isolate D46 (on plasmid pD46-4). C. Representation of the RI-PER-7 in plasmid P7774-unnamed 1. D. Representation of the transposon harboring aminoglicosides resistance genes *aac*(3'')-*IIa* and *aac*(6')-*Ian*.
